# Association between Chronic Interstitial Cystitis and Herpes Zoster

**DOI:** 10.3390/ijerph17072228

**Published:** 2020-03-26

**Authors:** Chao-Yu Hsu, Cheng-Li Lin, Chia-Hung Kao

**Affiliations:** 1 Department of Medical Education and Research, Puli Christian Hospital, Puli 545, Taiwan; hsuchaoyu66@yahoo.com; 2 Department of Family Medicine, Puli Christian Hospital, Puli 545, Taiwan; 3 Department of Optometry, Central Taiwan University of Science and Technology, Taichung 40601, Taiwan; 4 Center for General Education, National Taichung University of Science and Technology, Taichung 404, Taiwan; 5 The General Education Center, Chaoyang University of Technology, Taichung 413, Taiwan; 6 Department of General Education, National Chin-Yi University of Technology, Taichung 41170, Taiwan; 7 Center for General Education, National Chi Nan University, Puli 54561, Taiwan; 8 Rural Generalist Program Japan, genepro, Asahi Shi 289-2505, Japan; 9 Management Office for Health Data, China Medical University Hospital, Taichung 40447, Taiwan; orangechengli@gmail.com; 10 School of Medicine, College of Medicine, China Medical University, Taichung 40447, Taiwan; 11 Graduate Institute of Biomedical Sciences, China Medical University, Taichung 40447, Taiwan; 12 Department of Nuclear Medicine and PET Center, China Medical University Hospital, Taichung 40447, Taiwan; 13 Department of Bioinformatics and Medical Engineering, Asia University, Taichung 40447, Taiwan; 14 Center of Augmented Intelligence in Healthcare, China Medical University Hospital, Taichung 40447, Taiwan

**Keywords:** chronic interstitial cystitis, herpes zoster, depression

## Abstract

Objectives: Herpes zoster (HZ) infection has been associated with disease burdens such as infection and depression. However, the relationship between chronic interstitial cystitis (CIC) and HZ is unknown. This study investigated HZ risk in patients with CIC. Patients and Methods: The Longitudinal Health Insurance Database, which is a subset of the Taiwan National Health Insurance Research Database, was used in the study. The case cohort consisted of patients with newly diagnosed CIC between 2000 and 2012. Each patient with CIC was matched to four controls by age and index year. All participants were traced from the index date to HZ diagnosis, and loss to follow-up or death, or to the end of the study (31 December 2013). Results: A total of 1096 patients with CIC and 4384 controls were enrolled. The incidence rate of HZ in patients with CIC was 10.8 per 1000 person-years, whereas that for controls was 7.25 per 1000 person-years. HZ risk for the case cohort was 1.48 times that for the control cohort. Among participants aged ≤49 years, patients with CIC had a 1.91-fold-increased HZ risk compared to those without CIC. Conclusion: Patients with CIC had a higher risk of HZ than those without CIC. CIC should not be ignored, particularly in young adults.

## 1. Introduction

Chronic interstitial cystitis (CIC) is also known as bladder pain syndrome. According to the definition given by the National Institute of Diabetes and Digestive and Kidney Diseases, it is a chronic condition that causes a painful urinary syndrome. The cause of CIC is still poorly understood. The syndromes of CIC are urgency, nocturia, and pain in the pelvic area [1].

CIC is more prevalent in women (52–500 cases per 100,000 women) than in men (8–41 cases per 100,000 men) [2]. Patients with CIC have a relatively high prevalence of depression [3]. A multimodal approach to treatment—that features, for example, oral medication administration and minimally invasive techniques—is recommended. However, to date, CIC remains a controllable but not curable disease [4].

Herpes zoster (HZ) is caused by the reactivation of the varicella-zoster virus, and is characterized by painful vesicular rashes with a dermatomal distribution. Through a systematic review, Kawai et al. observed that HZ incidence was 3–5 per 1000 person-years in North America, Europe, and the Asia Pacific region. Furthermore, they found that HZ incidence increased with age, with 6–8 and 8–12 cases per 1000 person-years at 60 and 80 years old, respectively [5]. Postherpetic neuralgia is a painful complication. It may occur after the acute stage, affecting from 5% to 30% of patients [5].

The relationship between HZ and disease burdens, such as infection and depression, has been identified [6,7,8]. However, the association between CIC and HZ is unknown. CIC and CIC-related syndrome could be a stressor for suffering individuals, and hence, a relationship between CIC and HZ might exist. This study investigated HZ risk in patients with CIC.

## 2. Materials and Methods

### 2.1. Data Source

The Taiwanese government initiated a National Health Insurance (NHI) program in 1995. Most residents of Taiwan are included in the program. The medical claims of insured patients are recorded in the National Health Insurance Research Database (NHRID). In this study, we used the Longitudinal Health Insurance Database, which is a subset of the NHRID and contains the medical information, such as outpatient visits, hospitalization records, and medication usage, of 1 million randomly selected beneficiaries from the NHI program. The diagnostic codes, such as CIC or HZ, were defined according to the International Classification of Diseases, Ninth Revision, Clinical Modification (ICD-9-CM). All identification data were encrypted for ethical reasons.

### 2.2. Study Population

Patients with newly diagnosed CIC (ICD-9-CM: 595.1) between 2000 and 2012 were assigned to the case cohort. Patients aged less than 20 years or with a history of HZ were excluded. Each CIC patient was matched with 4 controls according to age and index year. The index year of the case cohort was the year of CIC diagnosis, and that of the control cohort was randomly assigned. We traced all participants from the index date to HZ diagnosis, and loss to follow-up or death, or to the end of the study (31 December 2013).

### 2.3. Outcome Measurement and Comorbidities

The primary event of this study was HZ (ICD-9-CM: 053). Chronic kidney disease (ICD-9-CM: 585, 586), obesity (ICD-9-CM: 278), diabetes (ICD-9-CM: 250), coronary artery disease (CAD; ICD-9-CM: 410–414), depression (ICD-9-CM: 296.2, 296.3, 300.4, 311), and cancer (ICD-9-CM: 140–208) were considered comorbidities.

### 2.4. Statistical Analysis

To compare the distribution of the baseline characteristics of the two cohorts, we used the chi-square test for categorical variables and the *t* test for continuous variables. The hazard ratio was estimated using the Cox proportional hazard regression model and then adjusted for age, sex, and CAD in a multivariable model. We assessed the cumulative incidence curve using the Kaplan–Meier method and examined it by using the log-rank test.

## 3. Results

A total of 1096 patients with CIC and 4384 controls were enrolled in this study. They were observed for approximately 6 years. Table 1 presents the demographic variables and comorbidities of the two cohorts. The distributions of age and sex in the two groups were similar after matching. A higher proportion of patients with CIC had diabetes, CAD, depression, and chronic kidney disease than controls.

**Figure 1:**
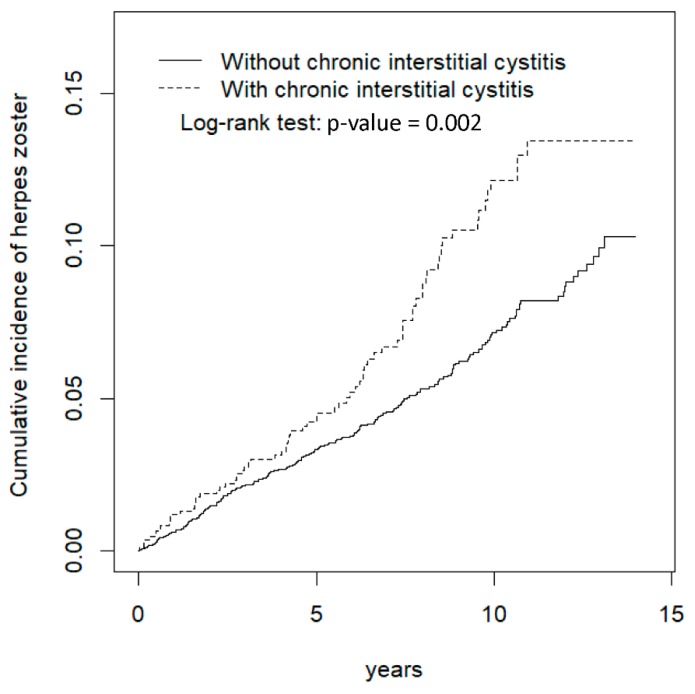
Cummulative incidence comparison of herpes zoster for patients with (dashed line) or without (solid line) chronic interstitial cystitis.

Figure 1 shows that the cumulative incidence of HZ in participants with CIC was significantly higher than that in those without CIC. The incidence rate of HZ for patients with CIC was 10.8 per 1000 person-years and that for controls was 7.25 per 1000 person-years (Table 2). HZ risk for the case cohort was 1.48 times that of the control cohort. Compared with patients aged ≤49 years, patients aged 50–64 and >65 years had an adjusted hazard ratio (aHR) of 2.86 (95% confidence interval [CI] = 2.00, 3.50) and 2.92 (95% CI = 2.11, 4.04), respectively.

The effects of CIC on HZ depending on age, gender, and the presence of comorbidities are listed in Table 3. Among the participants aged ≤49 years, patients with CIC had a 1.91-fold (95% CI = 1.24, 2.94) higher risk of HZ than those without CIC. The effect of CIC on HZ was significant in women (aHR = 1.46) and participants without any comorbidities (aHR = 1.48).

## 4. Discussion

To our knowledge, this is the first population-based study to identify the association between CIC and HZ. Patients with CIC were found to have a higher risk of HZ than those without CIC, particularly in those aged ≤49 years.

The prevalence rate of CIC is between 2% and 17.3% in the general population [9]. The majority of the patients with CIC were women (approximately 90%) [10]. The relationship between CIC and sex is still poorly understood, and several studies have attempted to identify the association. Rudick et al. reported that CIC–sex association is related to hormones. In an animal study, they found that murine neurogenic cystitis is mediated by a sex-specific response to mast cells [10]. Tyagi et al. reported that after cyclophosphamide administration, inflammation and cytotoxicity observed in the bladders of rats; these were accompanied by sex-related differences in nitric oxide reaction products and transforming growth factor-beta1 in the urine [11]. These studies can serve as references for further studies on sex-related difference in CIC.

An association between CIC and depression has been identified. Cepeda et al. reported that compared with 0.06% of the general population, 0.13% of patients with depression developed CIC within 2 years. The incidence of CIC is higher in patients with depression than in the general population [12]. CIC often occurs in patients with depression, and vice versa. Patients with CIC are at risk of depression. Using Beck’s Depression Inventory II Questionnaire, Goldstein et al. found a high prevalence of depression among women with CIC; 69% of the women with CIC scored ≥14, which indicated depression [13]. Chuang et al. found that the incidence of depression in patients with CIC was significantly higher than that in matched controls, with 101.0 and 42.2 per 10,000 person-years, respectively [14]. Women with CIC were 3.97 times more likely to develop depression than controls [15], and depression was more prevalent in women with CIC than in general population [16].

Women with CIC and depression have a high likelihood of experiencing abdominal or bladder pain [15]. Rabin et al. found that the upper limit of pain was higher in women with CIC than in patients with other chronic pain. Women with CIC experienced considerable pain and depression, and depression severity is associated with pain [16]. Depression and significant pain are associated with suicidal ideation among women with CIC. Tripp et al. reported that depression and pain are predictive factors for suicidal ideation in women with CIC [17]. Suicidal ideation was observed in 6% of healthy controls and 23% of patients with CIC who were followed for 2 weeks [17]. Because suicidal ideation among patients with CIC is high, Goldstein et al. suggested that patients with CIC should be screened for depression.

A strong association exists between depression and HZ. Chen et al. found that compared with controls, patients with HZ had a considerably higher incidence of major depression (2.2% vs. 1.4%) and any depressive disorder (4.3% vs. 3.2%) [18]. Patients with HZ had a high incidence of depression, and vice versa. Two population-based studies have reported that patients with depression have a high HZ. Liao et al. reported that the incidence of HZ was 4.58 per 1000 person-years among patients with depression, whereas it was only 3.54 per 1000 person-years among controls [8]. Choi et al. found that the incidence rate of HZ was considerably higher in patients with depression than in controls (6.8% vs. 6.3%) [19]. Liao et al. and Choi et al. have found that patients with depression were 1.11 and 1.09 times more likely to develop HZ than those without depression. They reported similar results of HZ risk. According to both studies, the highest risk of HZ among patients with depression was found in middle-aged patients [8,19]. Our results showed a higher prevalence of depression in the CIC group (Table 1), and a weakness of association between depression and HZ (Table 2); we considered that CIC must be a stressful factor for HZ development among suffering individuals and depression is a weak mechanism. Our study population was patients with CIC—the different study results may due to the different study population.

Evidence indicates that HZ risk increases with age due to the decrease in immune system robustness. We proved this relationship (Table 2), and we found the risk of HZ infection was 2.65 and 2.93 times higher among patients aged 50 to 65 years and more than 65 years, respectively, compared with patients aged less than 50 years. However, among the patients with CIC, the highest HZ risk was among those aged <50 years. CIC itself and CIC-related symptoms such as depression and pain were health burdens. Having CIC puts a patient at high risk of developing HZ, particularly among young adult patients. Because our results showed that the relationship between depression and HZ was weak among the patients with CIC, we identified that the association between CIC and HZ was obvious. Of course, several stressful factors may associate with CIC. In the text, we described pain; CIC-related syndromes such as pain may also be a stressful factor.

This study analyzed a subset of the Taiwanese NHIRD. The NHIRD has a large sample size and is highly representative of the population. This study, being retrospective, has several limitations. First, biases regarding CIC or HZ diagnosis may exist among medical specialists due to the diagnostic criteria. However, all insurance claims are sent to the National Health Insurance Administration and are reviewed by reimbursement experts. Therefore, diagnostic codes assigned are reliable. Second, disease severity is not recorded in the NHIRD. The severity of IC and HZ might cause different outcomes. Third, lifestyle is not recorded in the NHIRD. A balanced diet and moderate exercise are good for health and might influence the immunity of participants. Despite these potential limitations, the large sample size of this study yielded a powerful statistical analysis. Participants with CIC were found to have a higher risk of developing HZ than those without CIC.

## 5. Conclusions

Patients with CIC have a higher HZ risk than healthy individuals. Hence, CIC burden should not be ignored, particularly in young adults.

## Figures and Tables

**Table 1:** Demographic characteristics and comorbidities in cohorts with and without chronic interstitial cystitis.

Variable	Chronic Interstitial Cystitis		*p*-Value
	No	Yes	
	*n* = 4384	*n* = 1096	
**Age, year**			0.99
≤49	2432 (55.5)	608 (55.5)	
50–64	1116 (25.5)	279 (25.5)	
65+	836 (19.1)	209 (19.1)	
Mean ± SD ^†^	48.8 ± 16.9	49.2 ± 16.7	0.001
**Sex**			0.99
Female	3560 (81.2)	890 (81.2)	
Male	824 (18.8)	206 (10.8)	
**Comorbidity**			
Diabetes	259 (5.91)	84 (7.66)	0.03
CAD	573 (13.1)	219 (20.0)	<0.001
Depression	211 (4.81)	159 (14.5)	<0.001
Chronic kidney disease	65 (1.48)	25 (2.28)	0.06
Obesity	68 (1.55)	18 (1.64)	0.83
Cancer	106 (2.42)	34 (3.10)	0.20

Chi-Square Test; ^†^: *T*-Test; CAD denotes coronary artery disease.

**Table 2:** The incidence and risk factors for herpes zoster.

Variable	Event	PY	Rate ^#^	Crude HR (95% CI)	Adjusted HR ^&^ (95% CI)
**Chronic interstitial cystitis**					
No	212	29,240	7.25	1.00	1.00
Yes	77	7113	10.8	1.50 (1.16, 1.95) **	1.48 (1.14, 1.92) **
**Age, year**					
≤49	95	21,261	4.47	1.00	1.00
50–64	112	9150	12.2	2.76 (2.10, 3.63) ***	2.65 (2.00, 3.50) ***
65+	82	5943	13.8	3.15 (2.34, 4.24) ***	2.92 (2.11, 4.04) ***
Sex					
Female	249	29,489	8.44	1.45 (1.04, 2.03) *	1.33 (0.95, 1.86)
Male	40	6865	5.83	1.00	1.00
**Comorbidity**					
**Diabetes**					
No	270	34,357	7.86	1.00	1.00
Yes	19	1996	9.52	1.23 (0.77, 1.96)	
**CAD**					
No	222	31,457	7.06	1.00	1.00
Yes	67	4896	13.7	1.95 (1.48, 2.56) ***	1.17 (0.87, 1.58)
**Depression**					
No	272	34,356	7.92	1.00	1.00
Yes	17	1998	8.51	1.12 (0.68, 1.83)	
**Chronic kidney disease**					
No	284	35,914	7.91	1.00	1.00
Yes	5	439	11.4	1.46 (0.60, 3.54)	
**Obesity**					
No	286	35,929	7.96	1.00	1.00
Yes	3	424	7.07	0.93 (0.30, 2.91)	
**Cancer**					
No	286	35,609	8.03	1.00	1.00
Yes	3	744	4.03	0.51 (0.16, 1.58)	

Rate ^#^, incidence rate, per 1000 person-years; Crude HR *, relative hazard ratio; Adjusted HR ^&^ multivariable analysis including age, sex, and comorbidities of CAD; * *p* < 0.05, ** *p* < 0.01, *** *p* < 0.001.

**Table 3:** Incidence of herpes zoster by age, sex and comorbidity and Cox model measured hazards ratio for patients with chronic interstitial cystitis compared those without chronic interstitial cystitis.

Variables	Chronic Interstitial Cystitis						Crude HR * (95% CI)	Adjusted HR ^&^ (95% CI)
	No			Yes				
	Event	PY	Rate ^#^	Event	PY	Rate ^#^		
**Age, years**								
≤49	65	17,109	3.80	30	4152	7.22	1.92(1.25, 2.96) **	1.91(1.24, 2.94) **
50–64	82	7342	11.2	30	1808	16.6	1.49(0.98, 2.27)	1.50(0.98, 2.29)
65+	65	4790	13.6	17	1152	14.8	1.09(0.64, 1.86)	1.03(0.60, 1.77)
**Sex**								
Female	183	23,698	7.72	66	5790	11.4	1.48(1.12, 1.96) **	1.46(1.10, 1.93) **
Male	29	5542	5.23	11	1323	8.32	1.60(0.80, 3.20)	1.63(0.81, 3.28)
**Comorbidity ^§^**								
No	150	23,198	6.47	44	4785	9.20	1.42(1.02, 1.99) *	1.48(1.05, 2.07) *
Yes	62	6042	10.3	33	2328	14.2	1.38(0.90, 2.10)	1.51(0.99, 2.31)

Rate ^#^, incidence rate, per 1000 person-years; Crude HR *, relative hazard ratio; Adjusted HR ^&^: multivariable analysis including age, sex, and comorbidities of CAD; ^§^ Individuals with any comorbidity of diabetes, CAD, depression, chronic kidney disease, obesity, and cancer were classified into the comorbidity group; * *p* < 0.05, ** *p* < 0.01, *** *p* < 0.001

## Abbreviations

HZ	Herpes zoster
CIC	chronic interstitial cystitis
NHIRD	National Health Insurance Research Database
ICD-9-CM	International Classification of Diseases, Ninth Revision, Clinical Modification
CI	confidence interval
HR	hazard ratio
